# Swin Transformer-based automatic delineation of the hippocampus by MRI in hippocampus-sparing whole-brain radiotherapy

**DOI:** 10.3389/fnins.2024.1441791

**Published:** 2024-10-11

**Authors:** Liang Li, Zhennan Lu, Aijun Jiang, Guanchen Sha, Zhaoyang Luo, Xin Xie, Xin Ding

**Affiliations:** ^1^Department of Radiotherapy, The Affiliated Hospital of Xuzhou Medical University, Xuzhou, China; ^2^Department of Equipment, Affiliated Hospital of Nanjing University of Chinese Medicine (Jiangsu Province Hospital of Chinese Medicine), Nanjing, China; ^3^Department of Radiation Oncology, Xuzhou Central Hospital, Xuzhou, China; ^4^HaiChuang Future Medical Technology Co., Ltd., Zhejiang, China

**Keywords:** hippocampus, whole brain radiotherapy, automatic segmentation, Swin Transformer, MRI

## Abstract

**Objective:**

This study aims to develop and validate SwinHS, a deep learning-based automatic segmentation model designed for precise hippocampus delineation in patients receiving hippocampus-protected whole-brain radiotherapy. By streamlining this process, we seek to significantly improve workflow efficiency for clinicians.

**Methods:**

A total of 100 three-dimensional T1-weighted MR images were collected, with 70 patients allocated for training and 30 for testing. Manual delineation of the hippocampus was performed according to RTOG0933 guidelines. The SwinHS model, which incorporates a 3D ELSA Transformer module and an sSE CNN decoder, was trained and tested on these datasets. To prove the effectiveness of SwinHS, this study compared the segmentation performance of SwinHS with that of V-Net, U-Net, ResNet and VIT. Evaluation metrics included the Dice similarity coefficient (DSC), Jaccard similarity coefficient (JSC), and Hausdorff distance (HD). Dosimetric evaluation compared radiotherapy plans generated using automatic segmentation (plan AD) versus manual hippocampus segmentation (plan MD).

**Results:**

SwinHS outperformed four advanced deep learning-based models, achieving an average DSC of 0.894, a JSC of 0.817, and an HD of 3.430 mm. Dosimetric evaluation revealed that both plan (AD) and plan (MD) met treatment plan constraints for the target volume (PTV). However, the hippocampal D_max_ in plan (AD) was significantly greater than that in plan (MD), approaching the 17 Gy constraint limit. Nonetheless, there were no significant differences in D_100%_ or maximum doses to other critical structures between the two plans.

**Conclusion:**

Compared with manual delineation, SwinHS demonstrated superior segmentation performance and a significantly shorter delineation time. While plan (AD) met clinical requirements, caution should be exercised regarding hippocampal D_max_. SwinHS offers a promising tool to enhance workflow efficiency and facilitate hippocampal protection in radiotherapy planning for patients with brain metastases.

## 1 Introduction

Whole-brain radiotherapy (WBRT) is an effective treatment for patients with brain metastases (Berghoff and Preusser, 2018). Prophylactic cranial irradiation (PCI) can significantly reduce the probability of brain metastasis and improve the overall survival rate of patients (Gondi et al., 2010). However, WBRT can cause hippocampal damage and cognitive disorders, with an incidence ranging from 50 to 90%. This often manifests as short-term memory impairment, decreased attention, and problem-solving abilities, seriously affecting the patient’s quality of life (Fike et al., 2009; Peters et al., 2016). With advancements in radiotherapy and growing emphasis on post-radiotherapy quality of life, hippocampal avoidance whole-brain radiotherapy (HA-WBRT) has been shown to significantly improve cognitive function in patients post-treatment. The Radiation Therapy Oncology Group (RTOG) 0933 phase II trial demonstrated that protecting the hippocampus could reduce the incidence of cognitive dysfunction to 7% (Gondi et al., 2014). Subsequently, the NRG Oncology CC001 phase III trial confirmed these findings. Notably, the results showed that WBRT combined with memantine for hippocampal protection resulted in superior cognitive preservation in adult patients with brain metastases, compared to WBRT with memantine but without hippocampal protection. Importantly, there was no significant difference in intracranial progression-free survival (PFS) or overall survival (OS) (Brown et al., 2020). Therefore, protecting the hippocampus during midbrain radiotherapy for brain tumor patients can mitigate memory and cognitive impairment, consequently enhancing the overall quality of life.

According to RTOG 0933, outlining the hippocampus on axial T1-weighted MR images is essential (Gondi et al., 2014). However, the hippocampus is a complex structure, and accurate delineation is crucial for effective radiation treatment planning and minimizing radiation-related side effects (Mukesh et al., 2012; Walker et al., 2014). Additionally, the hippocampus is situated between the thalamus and the medial temporal lobe of the brain. In magnetic resonance imaging, the gray matter intensity of the hippocampus is very similar to that of surrounding structures like the amygdala, caudate nucleus, and thalamus, with no distinct boundary, making delineation difficult. Currently, the method of hippocampal delineation is mainly based on the anatomical expertise of the doctor, who refers to the patient’s MR images to outline the CT images. The accuracy of this approach depends on the registration precision between MR and CT images, as well as the physician’s proficiency and anatomical knowledge. Significant variability exists between the delineation results of different doctors. Therefore, improving the accuracy, efficiency, and standardization of hippocampal delineation is a key step in reducing the risk of radiation-induced brain injury. Automatic segmentation of the hippocampus from MR images remains a challenging task.

Deep learning approaches based on convolutional neural networks (CNNs) have been widely used due to their efficiency and accuracy (Erickson, 2021). In 2015, U-Net was first proposed, constructing a U-shaped deep network using encoders and symmetric decoders, achieving commendable performance in segmenting image edges (Ronneberger et al., 2015). Specifically, U-Net employs an encoder to extract low-level details and high-level semantic features from the image and utilizes a decoder to map the features back to the original size, thereby generating the segmented image. By establishing connections between the encoder and decoder, the features from corresponding layers in both components can be merged, which enhances the preservation of detailed information in the input image and improves segmentation outcomes. Given that CT and MR images are typically three-dimensional, a 3D U-Net (Çiçek et al., 2016) was designed. Building upon this framework, V-Net (Milletarì et al., 2016) integrates encoder information filtered by the decoder and adds a ResNet (He et al., 2016), which prevents gradient vanishing, accelerates network convergence, and achieves superior performance. Subsequently, several U-Net variants have been developed.

The CNN method typically utilizes deep convolution layers with an encoder-decoder architecture to capture global information. However, this process often relies on skip connections to compensate for the loss of shallow feature information. The convolution operation is inherently local due to the receptive field size, which limits its effectiveness, particularly in segmenting small objects (Fei et al., 2023).

In recent years, the Transformer has been widely adopted in medical image segmentation as an alternative architecture featuring a global self-attention mechanism. Models like TransFuse (Zhang Y. et al., 2021), MFSuse (Basak et al., 2022), TFormer (Zhang et al., 2023), and TransCeption (Azad et al., 2023) effectively capture edge information, enhance segmentation accuracy, and optimize network performance. However, these advancements come with increased parameters, computational complexity, and longer inference times. Despite their enhanced localization capabilities, these models still struggle to capture low-level details.

As a transformer-based model, the Vision Transformer (VIT) (Dosovitskiy et al., 2020) surpasses CNNs due to its global and long-range modeling capabilities. However, VIT’s computational efficiency is relatively low because it depends on a self-attention mechanism for feature extraction. Swin Transformer (Liu et al., 2021), a new variant of VIT, introduces a sliding window approach to constrain self-attention. This model integrates locality into multihead self-attention (MHSA) through local self-attention (LSA), embedding local details in the earlier layers. However, LSA’s performance is comparable to that of convolution and is inferior to dynamic filters. To improve this, an enhanced LSA module (ELSA) (Zhou et al., 2021) has been introduced to better capture local information. SwinBTS (Jiang et al., 2022), the first model to incorporate the ELSA Transformer module in brain tumor segmentation tasks, brings forward innovative approaches.

Building upon the success of the Swin Transformer and the detailed feature extraction capabilities of enhanced local self-attention (ELSA), we propose SwinHS, a novel neural network designed for the automatic segmentation of hippocampal MR images. SwinHS improves local detail extraction by incorporating a 3D ELSA Transformer module. Additionally, we introduce the spatial squeeze excitation (sSE) block, which allows feature maps to be more informative both spatially and across channels. The primary goal of this study was to develop an AI tool for automated hippocampal delineation, with a focus on validating the segmentation’s accuracy and clinical applicability, ultimately aiming to enhance workflow efficiency for clinicians.

## 2 Materials and methods

### 2.1 Data collection

The Ethics Committee (No. XYFY2023-KL155-01) approved the retrospective collection of 100 three-dimensional T1-weighted (3D-T1) MR images from patients who underwent hippocampus-protected whole-brain radiotherapy at the Department of Oncology and Radiology of Xuzhou Medical University between 2018 and 2023. The patient cohort included 61 males and 39 females, aged between 30 and 83 years, with a median age of 60 years. Any images depicting hippocampal tumor invasion were excluded prior to MRI. The images were obtained using a GE Discovery MR750 3.0 T (GE Healthcare, Milwaukee, WI, United States) magnetic resonance imaging system. The scanning protocol employed a slice thickness of 0.8 mm, a spatial resolution of ( 0.8×0.496×0.496 ) mm^3^. The sequence used was 3D BRAVO, with repetition time (TR) = 7 ms, echo time (TE) = 3 ms, flip angle (FA) = 12°, and the resulting images were exported and saved in DICOM files. For the study, 70 patients were allocated to the training set and 30 to the test set.

### 2.2 Manual delineation

In accordance with the hippocampus atlas contouring guidelines proposed by RTOG0933 (Gondi et al., 2014), a tumor radiotherapist who was thoroughly trained and experienced in hippocampal delineation manually outlined the hippocampus on 100 axial MR images using the Varian Eclipse 13.6 planning system (Varian Medical Systems, Palo Alto, CA, United States). To ensure accuracy, the delineation results were subsequently reviewed and, where necessary, adjusted by another expert in tumor radiotherapy. The two radiologists involved in this study have 13 and 14 years of experience respectively, ensuring a high level of expertise in interpreting the imaging data.

### 2.3 Model training and testing

**Figure 1:**
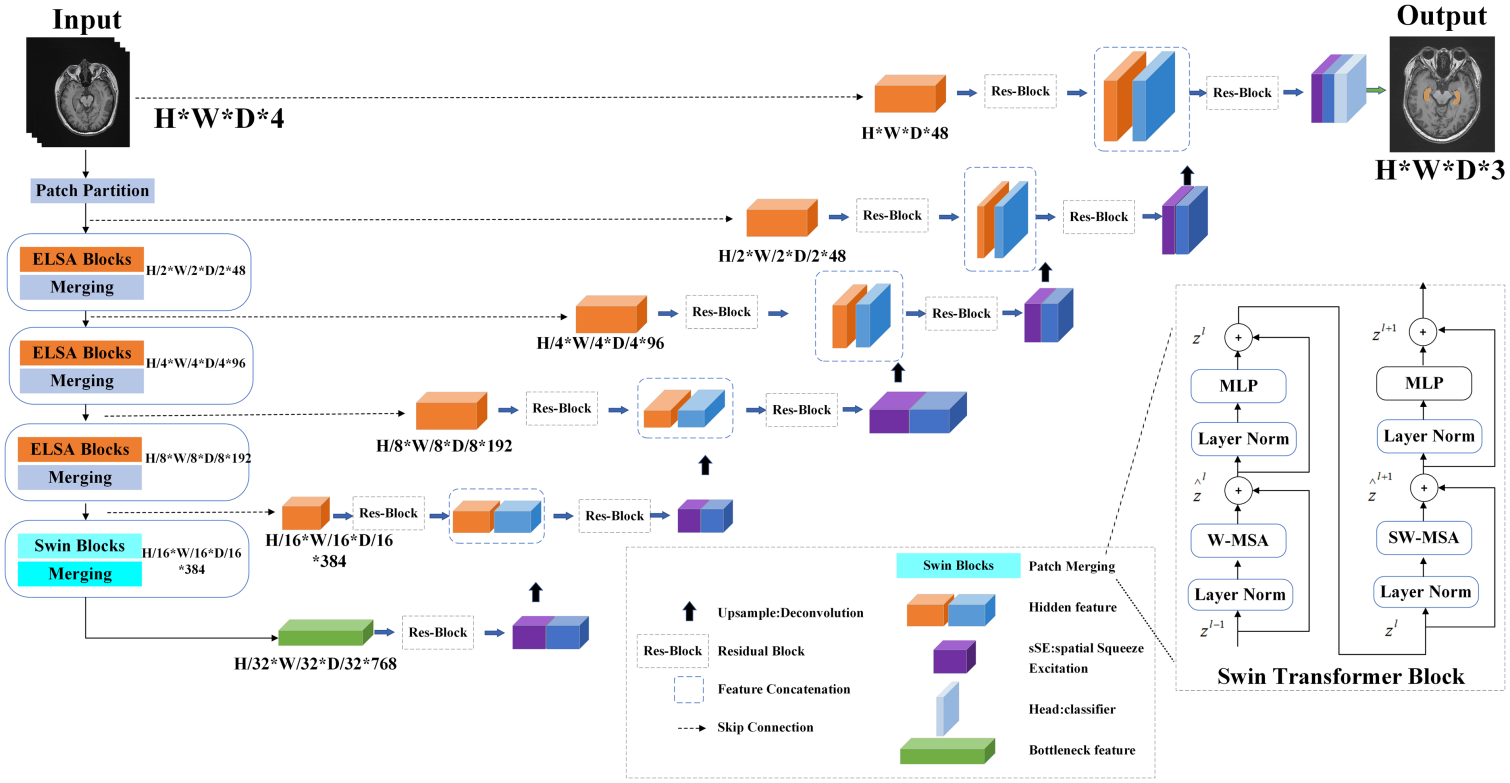
Overview of the model training process. The Transformer encoder includes three levels: each level includes two ELSA Transformer modules, and the next level includes two Swin Transformer modules. In the first level, the linear embedding layer is used to create a three-dimensional feature map. In the first, second and third stages, the ELSA module is used to extract very detailed feature information. Stage 4 uses the Swin Transformer to extract multiresolution features and the shift window mechanism to calculate self-attention. In these four stages, a slice merging layer is used to reduce the resolution of the feature by 2 times. On the right, the encoder is used to decode the extracted feature representations of the encoder through skip connections.

The overall architecture is illustrated in Figure 1. The input consists of a multimodal MR medical image $X\in R^{H\times W\times D\times C}$ , where the image size is H×W×D and *C* is the number of channels. These images are divided into non-overlapping patches, which are then passed to the transformer-encoder. The encoded features are subsequently processed through the ELSA module and the Swin Transformer module. Next, the feature representations are transmitted to the sSE CNN-decoder via skip connections at multiple resolutions, generating the final segmentation output. Each component of the proposed architecture is detailed in the following sections.

#### 2.3.1 Transformer encoder

Initially, we employ a 3D patch partition layer to segment medical images into nonoverlapping 3D patches with a volume of $\frac{H}{2}\times \frac{W}{2}\times \frac{D}{2}$ . Subsequently, these patches are projected into an embedding space with a dimensionality of *C*, enabling us to generate a feature map of size $\frac{H}{2}\times \frac{W}{2}\times \frac{D}{2}\times C$ .

#### 2.3.2 ELSA Transformer module

The ELSA Transformer module is employed to enhance local detailed feature extraction. ELSA introduces a novel local self-attention mechanism that outperforms both LSA and dynamic filters in the Swin Transformer. A key element of ELSA is Hadamard attention, which applies the Hadamard product to improve attention across neighboring elements while maintaining high-order mapping. In deep learning, it is commonly assumed that higher-order mappings offer stronger fitting capabilities. The low accuracy of some attention mechanisms may stem from their lower mapping order (Gondi et al., 2010), as the attention mechanism typically performs second-order mapping of the input, as described in Supplementary Formula 1.

**Figure 2:**
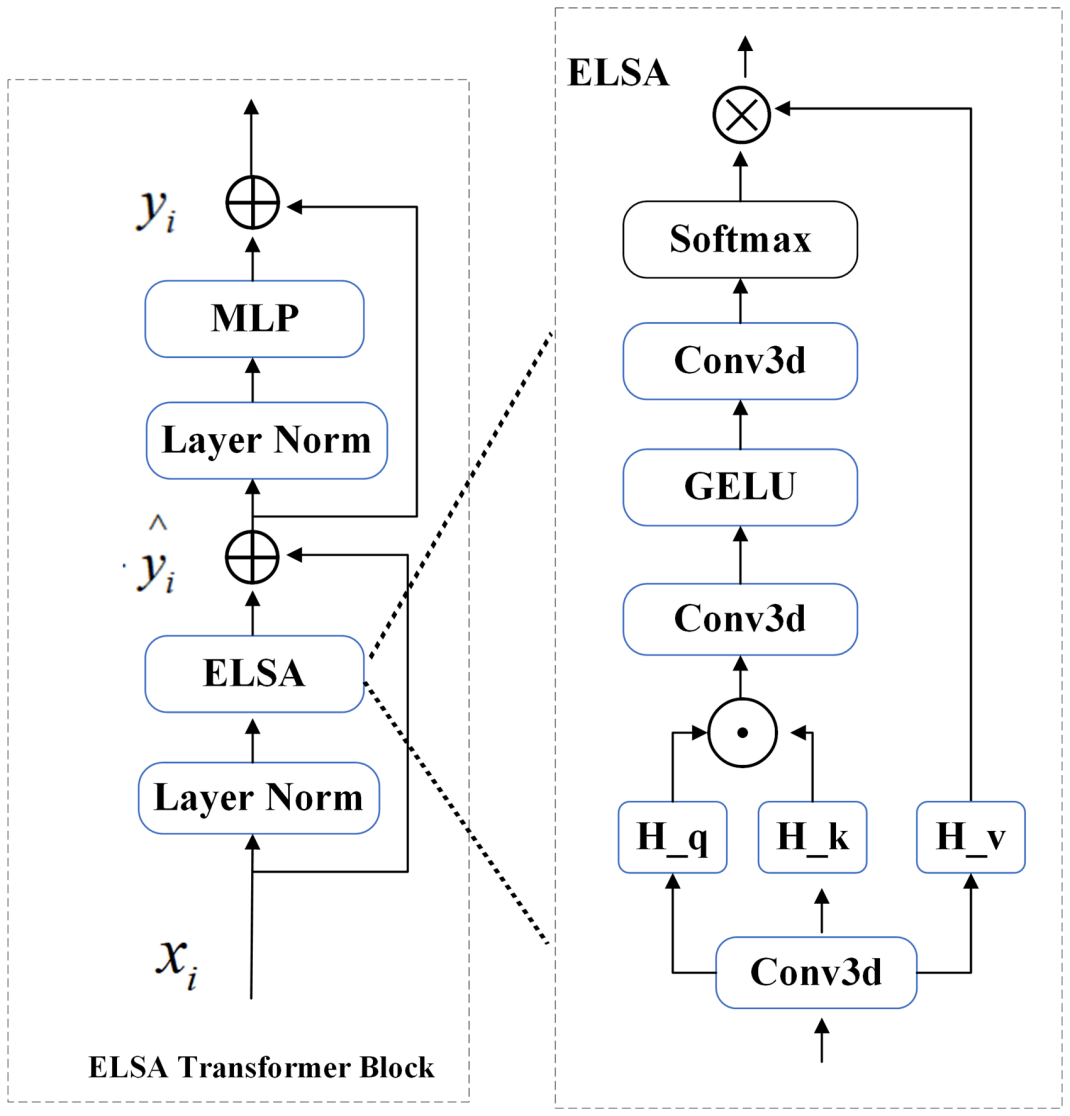
Structure of the ELSA Transformer block.

As illustrated in Figure 2, the ELSA Transformer module is derived by incorporating an identical MLP module subsequent to the attention structure in conjunction with the Transformer architecture, as depicted in Supplementary Formula 3.

#### 2.3.3 Swin Transformer module

The Swin Transformer is a hierarchical VIT that performs self-attention computations through an efficient shifted window partitioning scheme. This approach significantly reduces the number of parameters while enabling multiscale feature extraction with improved feature learnability. As shown in Figure 1, the Swin Transformer block in the architecture consists of a normalization layer (LN), window-based multihead self-attention module (MHSA), and multilayer perceptron (MLP).

#### 2.3.4 SSE CNN decoder

The decoder has the same depth as the encoder and is used to decode the feature representation of the extracted encoder. A skip connection is used between the encoder and decoder at each resolution. The output characteristics are reshaped to the size $\frac{H}{2^{i}}\times \frac{W}{2^{i}}\times \frac{D}{2^{i}}$ at each stage i ( i ∈ 0, 1, 2, 3, 4) of the encoder and the bottom, and then the residual block composed of two 3 × 3 × 3 normalized convolutional layers is input. Then, the sSE block is applied to the extracted features so that the feature map can provide more information both spatially and channelwise for image segmentation (Hu et al., 2018).

A linear transformation of the feature map is performed to enhance the dimension ( $\frac{H}{2}\times \frac{W}{2}\times \frac{D}{2}\times C$ ), which subsequently allows obtaining an output resolution similar to that of the image input, i.e., H×W×D resolution output. The final segmentation output is calculated by using a 1 × 1 × 1 convolutional layer and a sigmoid activation function.

For the model, the Adam optimizer is used for training, the initial learning rate is 1 × 10^−4^, and the weight is attenuated to 1 × 10^−5^. The default batch size is 50, and the default number of training iterations is 150. All experiments were performed using an Nvidia RTX2080Ti GPU.

### 2.4 Model evaluation

We use the Dice similarity coefficient (DSC), Jaccard similarity coefficient (JSC) and Hausdorff distance (HD) to evaluate the performance of our automatic delineation tool in the test set. The formulas are provided in the Supplementary material.

The DSC is the most commonly used metric for measuring the overlap between two contours, and its value is between 0 and 1. The larger the DSC value is, the greater the similarity between the two contour lines (Taha and Hanbury, 2015). Similarly, JSC compares the similarities and differences between finite sets (Eelbode et al., 2020). The larger the JSC is, the greater the sample similarity. HD describes the boundary similarity of 2 point sets by measuring the maximum distance of the closest pair of points. The smaller the HD is, the greater the coincidence degree between A and B, and the better the segmentation effect (Taha and Hanbury, 2015). In the special dosimetric evaluation reported by Gondi et al. (2014), deviations greater than 7 mm are considered unacceptable.

### 2.5 Comparison of model performance

To demonstrate the effectiveness of our proposed SwinHS, we compared its segmentation performance with that of four advanced deep learning-based methods: V-Net (Milletari et al., 2016), U-Net (Ronneberger et al., 2015), ResNet (He et al., 2016), and VIT (Chen et al., 2022). Then, each method was trained and tested on the same dataset using their respective frameworks. We evaluated the performance of SwinHS and the four other methods using DSC, JSC, and HD.

### 2.6 Radiotherapy planning and dosimetric evaluation

To evaluate the feasibility of applying hippocampal delineation via the SwinHS model in clinical practice, we conducted a study comparing simulated whole-brain radiotherapy plans for 10 randomly selected patients. We compared the differences in dosimetric distribution between two sets of radiotherapy plans: one using the manually delineated hippocampi and the other using the automatically delineated hippocampi by the SwinHS model. A large aperture CT simulator (Philips, Cleveland, OH, United States) was used to collect CT localization images of the patients’ head area, with a slice thickness of 1.5 mm. According to the RTOG 0933 report, the hippocampus is a low-signal gray matter structure that begins medially from the inferior horn of the lateral ventricle’s temporal horn and is bounded externally by the cerebrospinal fluid, typically forming a crescent shape. On the MR image, the contoured hippocampal tissue is expanded by 5 mm to create the hippocampus planning risk volume (HC-PRV). We registered the patient positioning CT image with the 3DT1 MR image on the MIM workstation (MIM Software Inc., Beachwood, OH). The delineated hippocampus and HC-PRV on the MR image were then mapped to the positioning CT, and the CT image was subsequently imported back to the treatment planning system (TPS) for the creation of the simulated radiotherapy plan. The target area was defined as follows: the patient’s whole brain tissue was the CTV, and the CTV was expanded by 3 mm and subtracted from the hippocampus to generate the planned target volume (PTV). The prescribed radiotherapy dose was 30 Gy, administered in 10 fractions. The goal of all plans was to cover at least 95% of the PTV with a 100% prescription dose.

We created two radiotherapy plans, plan (AD) and plan (MD), using the automatically contoured hippocampus (AD) and manually contoured hippocampus (MD) respectively, where the optimization parameters are identical. The radiotherapy plan was designed using the Varian VitalBeam (Varian Medical Systems, Palo Alto, CA, United States) 6 MV X-ray in FFF mode, utilizing the dynamic intensity-modulated radiotherapy (sIMRT) technique. A dose rate of 1,200 MU/min was applied across 9 noncoplanar irradiation fields. Dose calculations were performed with the Varian Eclipse 13.6 planning system, employing an anisotropic analytical algorithm with a spatial resolution of 2.5 mm.

Given that we used the manually delineated hippocampus as the reference standard for evaluating the accuracy of the automatically delineated hippocampus, our plan (AD) and plan (MD) evaluations were based on the manually delineated hippocampus to accurately reflect the hippocampal dose during radiotherapy. We compared the dose distribution differences between the radiotherapy plan (AD) and the radiotherapy plan (MD) using a dose-volume histogram (DVH) and assessed whether the relevant indicators in the radiotherapy plans met the dose limits outlined in the RTOG-0933 protocol (Gondi et al., 2015) and the NRG Oncology CC001 phase III trial (Brown et al., 2020). When administering whole-brain radiotherapy at 30 Gy/10 F, the indices included the following: (1) PTV: D_2%_ ≤37.5 Gy (D_2%_: the dose received by 2% of the PTV), D_98%_ ≥25 Gy (D_98%_: the dose received by 98% of the PTV), V30 Gy ≥90% (V_30 Gy_: the percentage of the PTV volume receiving 30 Gy). (2) Hippocampus: D_max_ ≤17 Gy (maximum dose), D_100%_ ≤10 Gy (D_100%_: the minimum dose received by the entire hippocampus). All treatment plans were designed by the same medical physicist with 5 years of experience in radiotherapy planning, and subsequently reviewed by other experts to ensure quality and adherence to clinical standards.

### 2.7 Statistical analysis

Paired t tests were conducted to compare the hippocampal volume, DSC, JSC and HD between the manual delineation group (MD) and the automatic delineation group (AD), as well as assess the differences in dosimetric parameters between the MD and AD plans. All the statistical analyses were performed using SPSS v22.0 software. A significance level of *p* < 0.05 was considered considered statistically significant.

## 3 Results

### 3.1 Patient characteristics

The characteristics of the patients in the training dataset and the test dataset are presented in Table 1. In the test dataset, a significant difference was observed between the hippocampus volumes in the manual delineation (MD) and automatic delineation (AD) groups, with a *p-*value of 0.019. Specifically, the hippocampus volume in the AD group was smaller than that in the MD group.

**Table 1:** Basic characteristics of the 100 patients.

	Total subjects (*n* = 100)	Training cohort (*n* = 70)	Testing cohort (*n* = 30)	
			MD	AD
Number of male patients (%)	61 (61)	43 (61.4)	18 (60)	
Number of female patients (%)	39 (39)	27 (38.6)	12 (40)	
Median age in years (range)	60 (30–83)	62.5 (33–81)	57.5 (30–83)	
Volume of hippocampus (±SD cm^3^)	4.02 ± 0.83	3.89 ± 0.85	4.32 ± 0.70	4.15 ± 0.67
*p*-value	-	-	0.019	

MD, manual hippocampus segmentation; AD, automatic segmentation; SD, standard deviation.

### 3.2 Performance comparison of the SwinHS models

We compared the segmentation results of five different models in the test dataset, as presented in Table 2, using DSC, JSC, and HD as evaluation metrics. The table demonstrates that our proposed model outperforms the other four models across all indicators. Specifically, the average DSC is 0.894 ± 0.017, the average JSC is 0.817 ± 0.020, and the average HD is 3.430 ± 0.245 mm.

**Table 2:** Results of different models.

	DSC	JSC	HD (mm)	*p*-value
SwinHS	0.894 ± 0.017	0.817 ± 0.020	3.430 ± 0.245	
VIT	0.891 ± 0.016	0.803 ± 0.016	3.959 ± 0.328	0.002
3D ResNet	0.871 ± 0.024	0.783 ± 0.022	4.730 ± 0.262	0.016
3D U-Net	0.845 ± 0.025	0.759 ± 0.019	6.895 ± 0.268	2.5 × 10^−4^
V-Net	0.778 ± 0.020	0.674 ± 0.023	7.785 ± 0.277	0.008

DSC, Dice similarity coefficient, JSC, Jaccard similarity coefficient; HD, Hausdorff distance.

**Figure 3:**
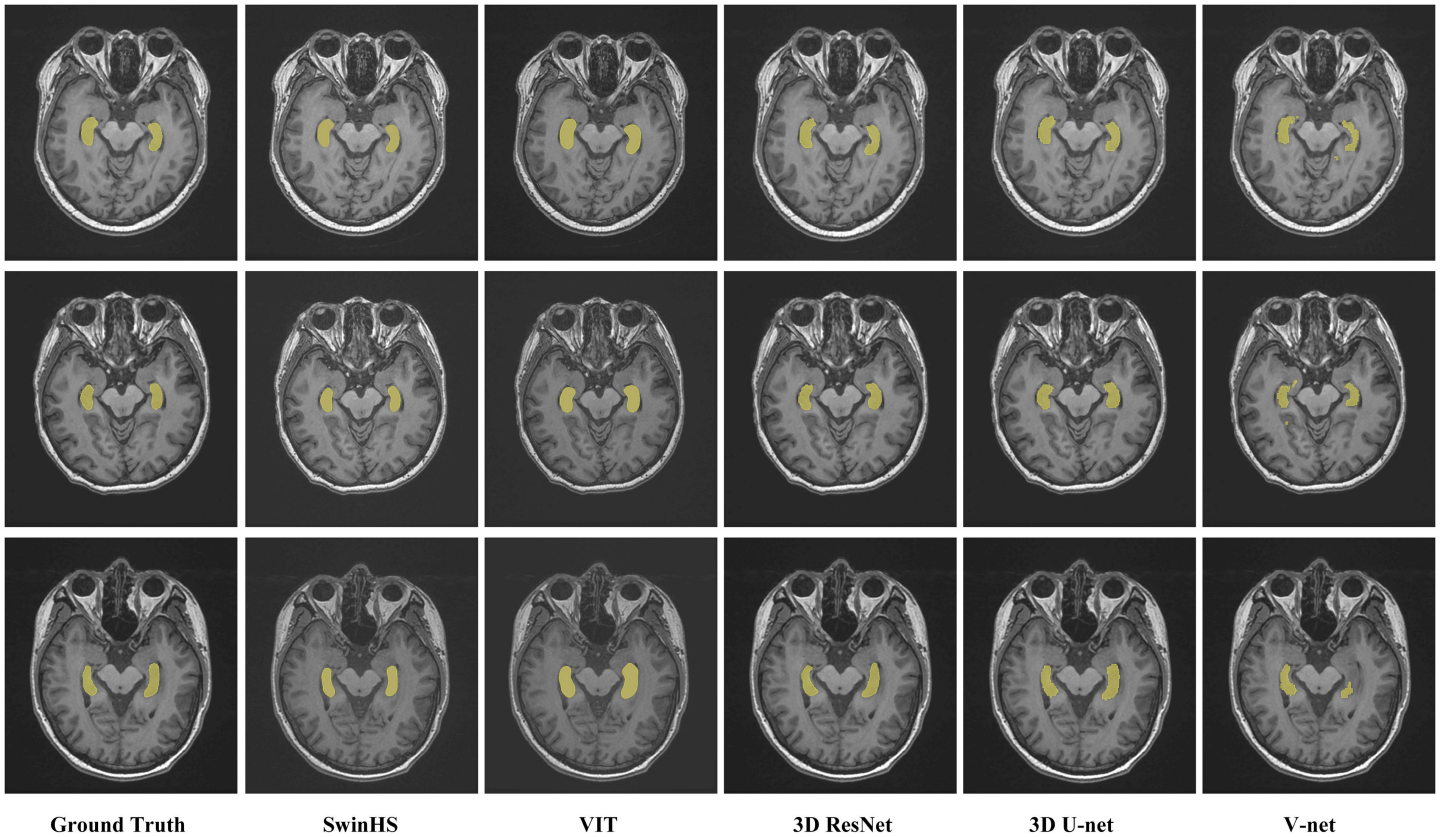
Representative cases of the proposed model and other model methods. The first column shows the contour of the hippocampus in the real MR image. Columns 2 to 6 show the results of the proposed model, VIT, 3D ResNet, 3D U-Net, and V-Net, respectively.

The segmentation results of the hippocampus at different levels between the proposed model and other models are visually compared, in Figure 3. The first column displays the actual manual segmentation of the hippocampus. From the second column onwards, it becomes evident that the proposed method shows greater consistency with the manual delineation of the hippocampus contour. In the third column, the contour delineated by VIT appears smooth but shows slight deviations from the actual delineation. The fourth and fifth columns reveal rough hippocampal contours delineated by 3D ResNet and 3D U-Net, respectively. Finally, in the sixth column, the hippocampal contour delineated by V-Net is depicted inaccurately and incompletely.

### 3.3 Dosimetric evaluation of the SwinHS model

According to the requirements of the RTOG0933 phase II trial (Gondi et al., 2015) and the NRG Oncology CC001 phase III trial (Brown et al., 2020), specific criteria must be met for radiotherapy planning. When administering whole-brain radiotherapy at 30 Gy/10 F, it is essential to ensure that the dose received by 2% of the planning target volume (PTV D_2%_) is ≤40 Gy and that the dose received by 98% of the PTV (D_98%_) is ≥25 Gy. Additionally, it is considered unacceptable if the volume of the PTV receiving 30 Gy (V_30 Gy_) exceeds 90%. Furthermore, for the hippocampus, it is imperative that the minimum dose (D_100%_) does not exceed 10 Gy, and the maximum dose (D_max_) remains under 17 Gy. In this study, radiotherapy plans were generated using both AD and MD hippocampus, denoted as plan (AD) and plan (MD), respectively. We utilized the MD hippocampus as the reference standard to assess the accuracy of the AD hippocampus. Subsequently, we compared the dose indicators and distribution differences between plan (AD) and plan (MD) based on MD hippocampus delineation.

**Figure 4:**
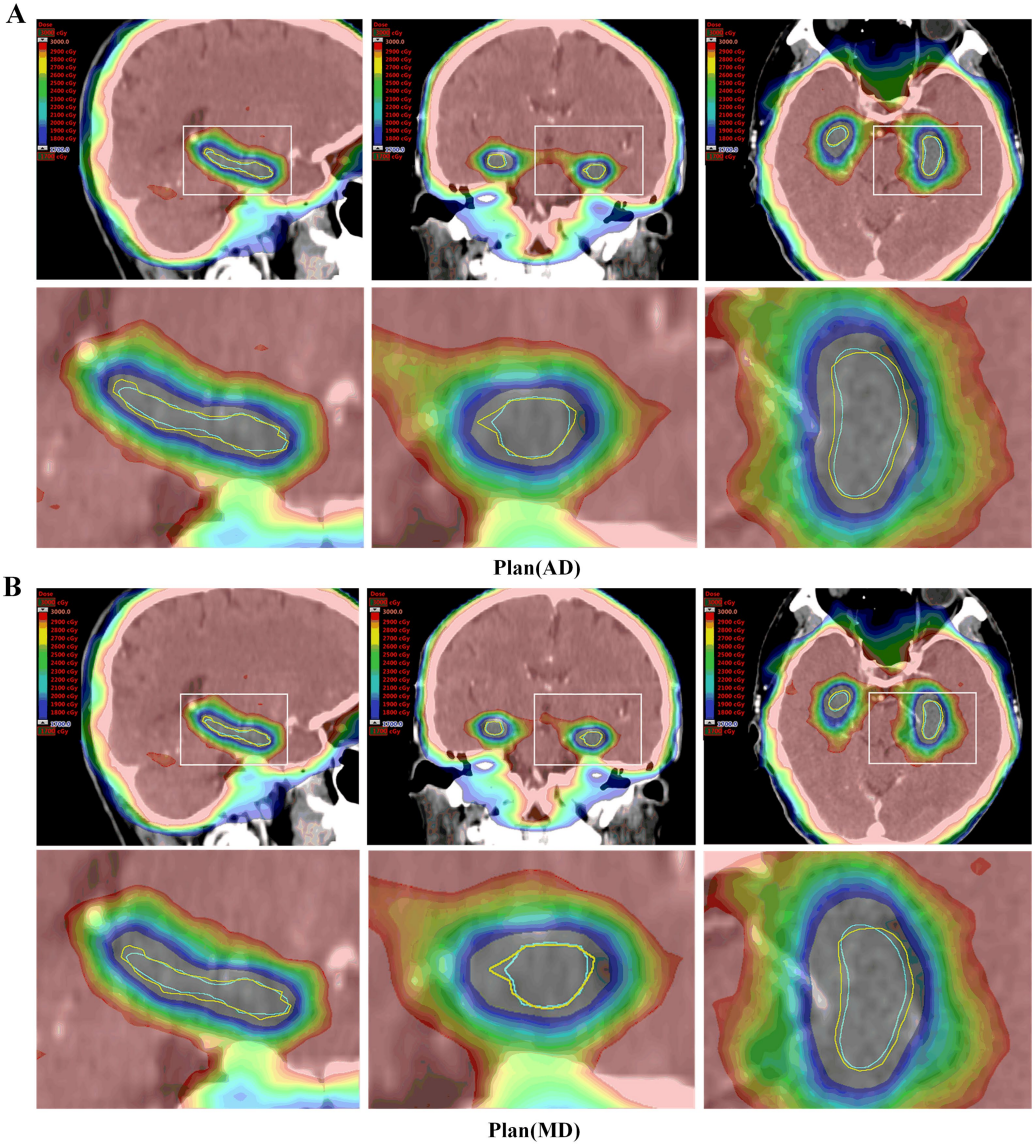
Dose distribution of representative patient plans (MD) and plans (AD). **(A)** Plan (AD) automatic delineation of the hippocampus-generated radiotherapy plan. **(B)** Plan (MD) manually outlines the radiotherapy plan generated by the hippocampus. The horse body was manually sketched (yellow line), and the hippocampus was automatically depicted (blue). Both plans were evaluated using manual delineation of the hippocampus. In the Plan (AD), a small portion of the manually delineated hippocampus was closer to a dose of 1700 cGy.

The representative patient dose distributions comparing automatic and manual hippocampus segmentation plans are shown in Figure 4. Plan (AD) was generated using automatically delineated hippocampus, while plan (MD) was based on hippocampus manually contoured by experienced clinicians. The contours in both plan (AD) and plan (MD) are the same; however, the manually contoured hippocampus serves as the reference standard for evaluating both plans. The volume of the automatically segmented hippocampus was smaller than that of the manually delineated hippocampus, resulting in the 17 Gy dose color brush being closer to the actual hippocampus in the automatic segmentation plan. As shown in Table 3, the dose indicators for PTV in both plan (AD) and plan (MD) met the treatment plan constraints recommended by the RTOG 0933 trial, with no significant differences observed between the two groups of plans. Regarding hippocampus dosimetry, although both plan (AD) and plan (MD) met acceptable variations, the hippocampus D_max_ in plan (AD) was significantly greater than that in plan (MD), with a notable difference (*p* < 0.001) at 1697.03 ± 11.02 cGy, approaching the limit of the 17 Gy constraint. Moreover, there was no significant difference in D_100%_ between the two groups (*p* = 0.236).

**Table 3:** Dosimetric comparison between plan (AD) and plan (MD).

		Plan (AD) (±SD)	Plan (MD) (±SD)	*p*-value
PTV	D_2%_ (cGy)	3353.25 ± 29.31	3352.71 ± 28.66	0.111
	D_98%_ (cGy)	2795.22 ± 21.75	2793.43 ± 22.32	0.172
	V_30Gy_ (%)	94.30 ± 0.73	94.30 ± 0.74	0.545
Hippocampus	D_max_ (cGy)	1697.03 ± 11.02	1474.25 ± 35.51	0.000
	D_100%_ (cGy)	997.22 ± 5.88	994.54 ± 7.13	0.236

D_max_, the maximum dose; V_30 Gy_, the volume of PTV getting 30 Gy; D_98%_, the dose received at 98% of PTV; D_2%_, the dose received at 2% of PTV; D_100%_, the dose to 100% of hippocampus.

### 3.4 Delineation time analysis

The median time required for automatic hippocampal delineation in the test group of 30 patients was 13.3 s (range: 11.7–14.9 s). This result was significantly shorter than the time required for manual delineation (MD) (*p* < 0.001), which was 786 s (range: 635–905 s).

## 4 Discussion

In this study, we employed a Swin Transformer-based neural network, SwinHS, to automatically segment hippocampal MR images. This network incorporated a 3D ELSA Transformer module to enhance local detailed feature extraction and a spatial squeeze excitation module (sSE) to integrate spatial and channel information. Four deep learning models, namely, V-Net, U-Net, ResNet, VIT, and the SwinHS network developed in this study, were trained and tested on the same dataset. Performance was evaluated using DSC, JSC, and HD metrics, and the dosimetric parameters of plan (AD) and plan (MD) were compared. The results demonstrated that the proposed model outperformed the other four models across all indicators, achieving a contouring effect more consistent with manual hippocampal delineation. The PTV of both the AD and MD plans met the constraints outlined in the RTOG 0933 treatment plan. However, the D_max_ of the hippocampus in the AD plans was significantly greater than that in the MD plans (*p* < 0.001), while the D_100%_ remained below 10 Gy, with no significant difference observed. These findings suggest that the automatic hippocampal segmentation method proposed in this study effectively extracts global features, accurately outlines hippocampal contours, and enhances hippocampal segmentation accuracy.

The RTOG-0933 protocol requires that the hippocampus be delineated on the patient’s high-resolution 3DT1-weighted MR image before HA-WBRT and then registered with the positioning CT for planning design (Gondi et al., 2015). In practice, manual segmentation by clinicians is time-consuming and labor-intensive, often leading to large segmentation errors. According to the RTOG-0933 test, nearly 7% of the hippocampus delineations by a doctor were deemed unqualified (Gondi et al., 2015). Additionally, manual segmentation of organs is highly subjective, with significant variation among doctors (Zhang J. et al., 2021). To address these challenges, scholars have conducted extensive research on accurate automatic hippocampal segmentation. Feng et al. (2020) used NeuroQuant software approved by the U.S. Food and Drug Administration to perform hippocampal segmentation on T1 MR in patients undergoing whole-brain radiotherapy. Among 100 patients, 99 underwent acceptable automatic hippocampal segmentation without manual intervention, with all plans meeting the PTV dose-volume target set by the NRG CC001 protocol. However, the segmentation technology of NeuroQuant is based on atlas-based registration (Fischl et al., 2002). Although this method provides accurate results and reduces manual effort, it requires significant computation and depends heavily on the choice of atlas, resulting in unstable segmentation performance. Wang et al. (2022) found that the deep learning (DL) model demonstrated superior segmentation performance, especially for smaller OARs, by comparing the differences between the multiatlas segmentation method and the deep learning method in the automatic segmentation (OARs) scheme of nasopharyngeal carcinoma risk organs. In recent years, deep learning methods based on convolutional neural network CNNs have been widely used in the field of medical images (Li and Shen, 2022). Among them, 3D U-Net-based models are widely used in medical image segmentation tasks (Li and Shen, 2022) (deep learning-based methods have been proposed, in which 3D U-Net was employed because it is widely used in medical image segmentation tasks). In addition, scholars have carried out in-depth research on automatic segmentation of the hippocampus. Lin et al. (2023) developed an improved 3D U-Net segmentation model. For CT images of 10 patients in the independent test set, the overall average DSC and 95% HD of the hippocampal contour were greater than 0.8 mm and less than 7 mm, respectively. All the plans met the RTOG 0933 standard. Porter et al. (2020) proposed the attention-gated 3D ResNet (proposed Attention-Gated 3D ResNet) network model to study the segmentation of the hippocampus on patients’ noncontrast CT, with Dice coefficients of 0.738/0.737 (left/right). However, these studies require strict registration of MR and CT images. The automatic segmentation tool for the hippocampus based on CT has made progress, but MRI is still the most reliable method for excluding the metastasis of the hippocampus. Hänsch et al. (2020) compared the automatic segmentation of the hippocampus based on a convolutional neural network (CNN) for MR and CT images and found that high-quality and anatomically accurate training contours can be generated on MR images and propagated to CT images to obtain optimal results. Therefore, Qiu et al. (2021) proposed a 3D U-Net model of multitask edge-aware learning for segmenting T1-weighted MR images of patients and obtained a Dice coefficient of 0.8483 ± 0.0036, an HD of 7.5706 ± 1.2330 mm, and an AVD of 0.1522 ± 0.0165 mm. In addition, Pan et al. (2021) proposed a CNN network structure based on 3D U-Net to segment the hippocampus on 3D-T1 MR images, with average DSC and AVD values of 0.86 and 1.8 mm, respectively. Encouraged and inspired by previous research, we propose a new automatic hippocampal segmentation model for 3DT1 MRI called SwinHS, which is based on the Swin Transformer. This model is designed to address the limitations of conventional CNN models and traditional Vision Transformers (VIT). Unlike these models, the Swin Transformer leverages a self-attention mechanism to capture long-range dependencies and context information across the entire input, significantly enhancing the model’s ability to understand complex spatial relationships in the hippocampal region. This global attention mechanism enables the model to accurately capture the spatial positioning of the hippocampus in MR images. Additionally, the network incorporates an enhanced version of local self-attention (ELSA) instead of LSA. The introduction of the Hadamard product in ELSA facilitates more efficient attention generation while preserving high-order mapping relationships (Ghazouani et al., 2024), thereby enhancing the extraction of local detailed features. Finally, the feature representation extracted by the decoder is passed through a multiresolution skip connection to the sSE CNN decoder, resulting in the final output segmentation map.

In traditional Vision Transformer (ViT) models, the input tokens have a fixed size, and the model operates at a fixed sampling rate of 16, which is effective for image classification tasks. However, for dense prediction tasks on high-resolution images, the computational complexity scales quadratically with image size, leading to significant computational costs (Fang et al., 2023). To address this limitation, we introduced a hybrid model that combines the strengths of Transformer architectures, which excel at capturing long-range dependencies, with the hierarchical structure of convolutional neural networks (CNNs), thereby reducing computational complexity while retaining the model’s ability to capture both global and local features.

When evaluating hippocampus segmentation performance, SwinHS demonstrated exceptional results across key performance metrics, including Dice similarity coefficient (DSC), Jaccard similarity coefficient (JSC), and Hausdorff distance (HD). Compared to other models, SwinHS achieved a high DSC of 0.894 and significantly reduced HD values. This improvement can be attributed to the Transformer architecture’s ability to capture global information while preserving local detail, making it highly effective for segmenting small and intricate structures like the hippocampus.

In terms of data processing efficiency, the innovative design of the SwinHS architecture significantly accelerates processing speed. While manual segmentation typically requires an average of 786 s, SwinHS completes the same task in just 13.3 s, drastically reducing the workload for clinical practitioners. Compared to other models, SwinHS combines the efficiency of deep learning with the adaptability of Transformers, speeding up the computation process without compromising accuracy. In hippocampus segmentation tasks, this translates to faster and more precise outcomes.

In conclusion, the high accuracy and efficiency of the Swin Transformer model are expected to positively impact the development of hippocampus avoidance whole-brain radiotherapy treatment plans in clinical practice. Accurate hippocampus segmentation is also expected to assist in the early detection and monitoring of diseases related to hippocampus atrophy, such as Alzheimer’s disease. Additionally, the model’s rapid processing capabilities can shorten the time patients wait for diagnostic results, improving the overall responsiveness of healthcare services.

On the other hand, our proposed model operates as a supervised learning model, which requires sufficient data and precise manual contours as training labels. Hence, to optimize the automatic segmentation performance of the hippocampus in hippocampus-shielded whole-brain radiotherapy, we deliberately excluded data from healthy adults and individuals with mental disorders. Instead, we compiled training datasets from relevant patient cohorts, a strategy also supported by Lei et al. (2023). Experimental findings indicate that this approach effectively leverages hippocampus guidance information from MR images of patients undergoing whole-brain radiotherapy, leading to improved hippocampal segmentation accuracy compared to traditional deep learning methods.

We employed the dynamic IMRT technique to compare the dosimetric differences between plan (AD) and plan (MD) in order to evaluate the clinical feasibility of the SwinHS model for automatic hippocampal segmentation. According to previous studies, VMAT technology provides excellent treatment plan quality for hippocampus-protected whole-brain radiotherapy (Lin et al., 2023) and is superior to IMRT in terms of efficiency (Soydemir et al., 2021). In a study by Jiang et al. (2019), conducted by our team, all treatment plans, including static IMRT, dynamic IMRT, VMAT, and TomoTherapy, met the RTOG 0933 dose standards for hippocampus protection in patients with limited brain metastases undergoing hippocampus-sparing whole-brain radiotherapy. However, compared to VMAT and TOMO, the average maximum doses delivered to the hippocampus using sIMRT and dIMRT were significantly lower. Despite this, the differences in the mean hippocampal dose among the sIMRT, dIMRT, VMAT, and TOMO groups were not statistically significant.

Additionally, studies have shown that flattening filter free (FFF) beams not only provide higher dose rates and reduce field scatter and electron contamination but also minimize normal tissue exposure outside the target area (Hrbacek et al., 2011; Ghemiş and Marcu, 2021; Ji et al., 2022). To enhance treatment effectiveness and reduce hippocampal dose, we opted for 9-field noncoplanar FFF-dynamic IMRT. According to our experimental results, the automatically delineated hippocampus had a smaller volume than the manually delineated one, as shown in Table 1. In most cases, the contours of the automatically delineated hippocampus closely matched the manual delineations, as illustrated in Figure 1. Additionally, since the manually delineated hippocampus was used to evaluate plan (MD), while the automatically delineated hippocampus was used to generate plan (AD), there was a notable difference in the average maximum dose (D_max_) to the hippocampus. Specifically, the average D_max_ in the manual plan (MD) was 1474.25 ± 35.51 cGy, while in the automatic plan (AD), it was 1697.03 ± 11.02 cGy. Despite this difference, both values remained within the permissible limits specified by RTOG 0933 for hippocampal doses. In terms of D_100%_, there was no statistically significant difference between the automatic and manual plans, with both remaining below 10 Gy. Additionally, no dosimetric differences were observed in the PTV between plan (AD) and plan (MD). This is consistent with our expectations, as the volume variation of the hippocampus is negligible compared to the overall PTV. According to the RTOG 0933 study, these findings are considered clinically acceptable.

Our model has some limitations and presents opportunities for future improvements. First, we focused solely on hippocampal segmentation, so future research should explore automatic segmentation of other normal tissues in MR images, such as the crystalline lens, eyeballs, and brainstem, to further enhance treatment efficiency. Second, in the RTOG 0933 trial, 15.85% of participants failed the centralized review due to fusion or hippocampal segmentation errors (Gondi et al., 2015). This highlights the need to explore accurate automatic MR and CT registration as a critical area for future development. Moreover, our model’s training process was limited by the use of a relatively small dataset. Given the variability in hippocampal shapes among patients, future research should involve larger, multicenter datasets to improve the model’s robustness and generalizability. While our model assists physicians in hippocampal segmentation, the importance of the hippocampus in whole-brain radiotherapy means it is not yet capable of fully automating the segmentation process. Post-segmentation review and calibration by clinicians remain essential.

## 5 Conclusion

In this paper, we propose a hippocampus segmentation method based on the Swin Transformer, which effectively captures global features and enhances segmentation accuracy. We believe this approach has the potential to significantly improve clinical treatment efficacy for patients undergoing whole-brain radiotherapy (WBRT), leading to better prognoses by reducing treatment-associated cognitive decline and improving overall outcomes.

## Data Availability

The raw data supporting the conclusions of this article will be made available by the authors, without undue reservation.
