# Supplementary material for: Swin Transformer-based automatic delineation of the hippocampus by MRI in hippocampus-sparing whole-brain radiotherapy
Source: Front Neurosci. 2024 Oct 11;18:1441791. doi: 10.3389/fnins.2024.1441791 (PMC11502472; doi:10.3389/fnins.2024.1441791)
Supplement: Supplementary file 1 [file Data_Sheet_1.docx]

Supplementary Material

# Supplementary Formulas

(1). Attention mechanism

$$y_{i}=Soft\max(f(x_{i}))x_{i}+x_{i}$$

where $x_{i}$ is the input feature map, $f(x_{i})$ is the convolution operation, and $y_{i}$ is the output feature map. The self-attention structure in the Transformer structure is the third-order structure of the input $x_{i}$ because it contains the second-order term $q_{i}k_{j}$ and combines it with the value $v_{i}$. This is the reason that the final result still needs to be improved. One solution is to use the ELSA with Hadamard attention (Zhou et al., 2021). Hadamard attention can be written as Formula (2):

$$\mathop{y}\limits^{\wedge} _{i}=Soft\max(f(H\_k\otimes H\_q))H\_v+x_{i}$$

where $f(x_{i})$ is a convolution operation, is the Hadamard product, and $H\_k$, $H\_q$ and $H\_v$ are the feature maps obtained via linear transformation of the input.

(2). ELSA Transformer module

$$y_{i}=MLP(LN(\mathop{y_{i}}\limits^{\wedge} ))+\mathop{y_{i}}\limits^{\wedge}$$

# Model evaluation

The DSC is the most commonly used metric for measuring the overlap between two contours, and its value is between 0 and 1. The DSC is defined as

$$DSC(A,B)=\frac{2\left|A\cap B\right|}{\left|A\right|+\left|B\right|}$$

where A is the manual contouring part and B is the automatic contouring part.

The JSC is defined as

$$J(A,B)=\frac{\left|A\cap B\right|}{\left|A\cup B\right|}=\frac{\left|A\cap B\right|}{\left|A\right|+\left|B\right|-\left|A\cup B\right|}$$

HD describes the boundary similarity of 2 point sets by measuring the maximum distance of the closest pair of points. The HD is defined as

$$HD(A,B)=\max(h(A,B),h(B,A))$$

$$h(A,B)=\max(\min\left\|a-b\right\|),\text{a}\in A,b\in B$$

where h(A, B) represents the maximum value of the minimum distance between each point in the A set and the B set.

# Supplementary Tables

**Table S1**. **Models with number of parameters**

|  | Numbers of parameters(M) | Batch size | Max epochs | Learning rate  (LR) | Optimizer |
| --- | --- | --- | --- | --- | --- |
| SwinHS | 140.1 | 16 | 50 | 0.0001 | adam |
| VIT | 324.5 | 32 | 100 | 0.0004 | adam |
| 3D ResNet | 147.21 | 16 | 100 | 0.0002 | adam |
| 3D U-net | 121.32 | 16 | 50 | 0.0003 | adam |
| V-net | 173.9 | 16 | 50 | 0.0003 | adam |
